# Impaired parent-reported health-related quality of life of underweight and obese children at elementary school entry

**DOI:** 10.1007/s11136-012-0211-x

**Published:** 2012-06-14

**Authors:** Amy van Grieken, Lydian Veldhuis, Carry M. Renders, Jeanne M. Landgraf, Remy A. Hirasing, Hein Raat

**Affiliations:** 1 Department of Public Health, Erasmus MC University Medical Center, P.O. Box 2040, 3000 CA Rotterdam, The Netherlands; 2 Institute of Health Sciences, Faculty of Earth and Life Sciences and EMGO Institute for Health and Care Research, VU University Amsterdam, Amsterdam, The Netherlands; 3 HealthActCHQ Inc., Cambridge, MA USA; 4 Department of Public and Occupational Health, EMGO Institute for Health and Care Research, VU University Medical Center, Amsterdam, The Netherlands

**Keywords:** Underweight, Overweight and obesity, Children, Health-related quality of life, CHQ-PF28

## Abstract

**Purpose:**

Examine the health-related quality of life of 5–6-year-old underweight, overweight and obese children.

**Methods:**

Our cross-sectional study included 3,227 parent–child dyads from the “Be active, eat right” study. Parents completed questionnaires regarding child and parental characteristics. Health-related quality of life of the child was measured using the Child Health Questionnaire Parent Form 28. Children were classified normal weight, overweight, obese, severely obese, and underweight according to the international age and gender BMI cutoff points. Bootstrap analyses were performed for general linear models corrected for potential confounding variables.

**Results:**

Severely obese children (β, −2.60; 95 % CI, −4.80 to −0.57, *p* < 0.01) and underweight children (β, −1.11; 95 % CI, −1.85 to −0.39, *p* < 0.01) had lower parent-reported scores on the physical summary scale. On the physical functioning profile scale parents of overweight and severely obese children also reported statistically significant lower scores (*p* < 0.05 and *p* < 0.01, respectively).There were no significant differences regarding the psychosocial summary scale scores between the different weight categories.

**Conclusion:**

Underweight and overweight children experience impaired health-related quality of life on the physical functioning domain. Physicians, teachers and parents should be aware of the possible negative impact on health-related quality of life in underweight and overweight 5–6-year-old children.

**Electronic supplementary material:**

The online version of this article (doi:10.1007/s11136-012-0211-x) contains supplementary material, which is available to authorized users.

## Introduction

According to a recent study, the global prevalence of overweight (including obesity) among preschool children (0–5 years) has increased from 4.2 % in 1990 to 6.7 % in 2010: If this trend continues, a prevalence of 9.1 % is projected in 2020 [1]. In the Netherlands, the prevalence of overweight and obesity among children and adolescents (age, 2–21 years) in 2009 was estimated at 12.8 % for boys and 14.8 % for girls [2].

Being overweight and/or clinically obese in childhood can have profound physical and psychosocial consequences (e.g., diabetes, hypertension and negative self-evaluation), which impact the everyday health-related quality of life for children and adolescents [3, 4]. Overweight adults have reported more limitations on the physical dimension of health-related quality of life compared to normal weight adults [5, 6]. Also, among overweight and obese adolescents and older school-aged children (10–18 years), decrements have been reported for both the physical and psychosocial dimensions of health-related quality of life [7–19]. However, the few studies that have reported on the association between health-related quality of life and being overweight among children (4–7 years) [20, 21] have reported contradicting results. Wake et al. [20] reported that health outcomes were similar for normal weight and obese children, whereas Skinner et al. [21] reported that parents of very obese children reported decreases in general health and limitations in activity.

The prevalence of underweight in children (2–6 years) was estimated as 13 % for boys and 12 % for girls in the Netherlands in 1997 [22]. An impact on health-related quality of life has been reported among underweight adults [23]. However, studies that have examined the association between health-related quality of life and being underweight in children and adolescents reported more mixed results [11, 20, 24–27].

At the age of 5–6 years when children enter elementary school, any limitations with regard to physical and psychosocial well-being and therefore health-related quality of life could impede a child’s start to his or her school career [28–30]. The aim of this study was to compare the health-related quality of life for 5–6-year-old children who are underweight, overweight, and obese relative to age-matched peers of normal weight by means of the Child Health Questionnaire Parent Form 28 (CHQ-PF28) [31, 32]. Our hypothesis was that the health-related quality of life of underweight, overweight and obese children would be lower (i.e., worse) relative to age-matched children of normal weight.

## Methods

### Study population

The present cross-sectional study used data obtained at enrollment in the “Be active, eat right” study, a cluster randomized controlled trial that aims to assess the effects of an overweight prevention protocol as described in detail elsewhere [33]. The Medical Ethics Committee of the Erasmus MC (University Medical Centre Rotterdam) approved the study protocol (reference number MEC-2007-163). A total of 13638 parents visiting one of the 44 participating Youth Health Care centers for their 5-year-old child’s regular preventive health check between 2007 and 2008 were invited to participate in the study. The Youth Health Care centers sent parents the regular invitation to come to the center for the child’s regular preventive health check; information on the study, an informed consent form and the first questionnaire on child and parental characteristics was included with the invitation. Of the parents who presented for their child’s health check, 64.4 % provided informed consent (*n* = 8,784) for participation in this study and 98.9 % (*n* = 8,683) of the parents with consent returned the first questionnaire. A second questionnaire, including the CHQ-PF28, was provided to all study participants visiting 22 Youth Health Care centers (*n* = 3,942) (these families were not eligible to receive an intervention) and to study participants with overweight or obesity in the remainder 22 Youth Health Care centers (*n* = 439) (these families were eligible to receive an intervention). The Youth Health Care centers were randomly assigned to either provide an intervention or not, in the context of the “Be active, eat right” study. Not all parents received the second questionnaire in order to minimize respondent burden. The response rate to the second questionnaire was 74.8 % (Figure 1 in the supplementary material shows a flow chart of the data collection process).

A study population of 3,227 children remained after removing records with missing data on child’s gender, weight, height or all CHQ-PF28 items (*n* = 51).

### Weight status of the child

Each child’s weight and height was measured by trained Youth Health Care professionals using standardized methods during the regular preventive health check [34]. Body mass index (BMI) was calculated by the researchers as weight in kilograms divided by height in meters squared. Children were categorized into one out of five weight categories according to their BMI as follows: underweight, normal weight, overweight (not (severe) obesity), obesity (not severe obesity) or severe obesity. The international age-specific and gender-specific BMI cutoff points were applied to categorize children into underweight, normal weight, overweight and obesity [35, 36]. Obese children were categorized into obese (not severely obese) and severely obese. There are currently no international BMI cutoff points for severe obesity for children, but based on recent literature [37] and sample size considerations, we used the following cutoff points to categorize severe obesity: for boys, BMI ≥ 20.00; for girls, BMI ≥ 21.00.

### Health-related quality of life

Health-related quality of life was assessed by means of the CHQ-PF28. There are 28 items on the CHQ-PF28 with four, five or six response options across eight multi-item scales and five single-item concepts. For the interpretation of the CHQ-PF28 scale scores and the psychometric properties of the CHQ-PF28 scales in the present study please see Table 1. As per the standardized developer instructions, the items from each of the scales were summed (some recoded/recalibrated) and transformed into 0 (worst possible score) to 100 (best possible score) scale [38]. From these “profiles,” it is also possible to compute a two-dimensional summary known as the physical and psychosocial summary scales [32]. Both summary scales were considered the main outcomes; the profile scales were analyzed in an exploratory way [39].

**Table 1:** CHQ-PF28 scales, number of items per scale, score interpretation and psychometric properties in the present study (*n* = 3,227)

CHQ-PF28 scales				Overall (*n* = 3,227)						
	Number of items	Description low score	Description high score	Missings	Mean [SD]	Median [IQR]	Score range	% min^b^	% max^b^	Cronbach’s α^c^
Physical summary^a^		Lower summary score represents lower physical health^a^	Higher summary score represents higher physical health^a^	101	56.8 [6.5]	58.4 [4.9]	−8.6–67.2	na	na	0.71^d^
Psychosocial summary^a^		Lower summary score represents lower psychosocial health^a^	Higher summary score represents higher psychosocial health^a^	101	53.0 [6.6]	53.8 [8.1]	12.2–66.2	na	na	0.83^d^
Physical functioning	3	Child is limited a lot in performing all physical activities, including self care, because of health	Child performs all types of physical activities, including the most vigorous, without limitations attributable to health	28	97.2 [11.0]	100.0 [0.0]	0–100	0.2	90.6	0.86
Role funct.-emo/behav.	1	Child is limited a lot in school work or activities with friends as a result of emotional or behaviour problems	Child has no limitations in schoolwork or activities with friends as a result of emotional or behaviour problems	26	97.5 [10.9]	100.0 [0.0]	0–100	0.2	94.2	na
Role funct.-physical	1	Child is limited a lot in school work or activities with friends as a result of physical health	Child has no limitations in schoolwork or activities with friends as a result of physical health	27	97.0 [11.9]	100.0 [0.0]	0–100	0.2	93.1	na
Bodily pain	1	Child has extremely severe, frequent, and limiting bodily pain	Child has no pain or limitations because of pain	28	85.7 [16.1]	80.0 [20.0]	0–100	0.6	44.7	na
General behavior	4	Child very often exhibits aggressive, immature, delinquent behaviour	Child never exhibits aggressive, immature, delinquent behaviour	17	71.8 [14.3]	71.3 [18.75]	0–100	0.0	2.6	0.69
Mental health	3	Child has feelings of anxiety and depression all of the time	Child feels peaceful, happy, and calm all of the time	28	81.7 [14.2]	83.3 [16.7]	0–100	0.0	20.5	0.64
Self-esteem	3	Child is very dissatisfied with abilities, looks, family/peer relationships, and life overall	Child is very satisfied with abilities, looks, family/peer relationships’ and life overall	22	82.1 [13.1]	75.0 [16.7]	0–100	0.1	24.3	0.82
Gen health perc.	4	Parent believes child’s health is poor and likely to get worse	Parent believes child’s health is excellent and will continue to be so	17	85.1 [15.7]	90.0 [18.8]	7.5–100	0.0	21.7	0.49
Parental-emotional	2	Parent experiences a great deal of emotional worry/concern as a result of child’s physical and/or psychosocial health	Parent doesn’t experience feelings of emotional worry/concern as a result of child’s physical and/or psychosocial health	20	89.2 [14.5]	100.0 [12.5]	0–100	0.1	51.3	0.38
Parental-time	2	Parent experiences a lot of limitations in time available for personal needs because of child’s physical and/or psychosocial health	Parent doesn’t experience limitations in time available for personal needs because of child’s physical and/or psychosocial health	27	95.2 [12.8]	100.0 [0.0]	0–100	0.2	82.7	0.56
Family activities	2	The child’s health very often limits and interrupts family activities or is a source of family tension	The child’s health never limits or interrupts family activities or is a source of family tension	24	89.2 [16.3]	100.0 [25.0]	0–100	0.2	59.6	0.70
Family cohesion	1	Family’s ability to get along is rated “poor”	Family’s ability to get along is rated “excellent”	63	73.6 [17.8]	85.0 [25.0]	0–100	0.1	14.8	na
Change in health	1	Child’s health is much worse now than 1 year ago	Child’s health is much better now than 1 year ago	34	56.2 [15.4]	50.0 [0.0]	0–100	0.2	7.9	na

Score interpretation: Reproduced with permission from the principal author Landgraf (page 38–39) [38]

*na* not applicable

^a^Physical and Psychosocial CHQ summary scores based on a factor-analytical model on U.S. population samples. A score of 50 represents the mean in the general U.S. population; scores above/below 50 are above/below the average in the general U.S. population [38]. Summary scales do not include the family activities, family cohesion and change in health scales

^b^% of respondent with the highest, respectively lowest possible CHQ-PF28 scale score (ceiling/floor)

^C^Average Cronbach’s α of the eight multi-item scales 0.77

^d^Cronbach’s α of the summary scales might be higher because the single-item scales (role func-emo/behav, role func-physical and bodily pain) could not be included

### Child and maternal characteristics

Information on child gender (male and female), age (years), ethnicity (Dutch, non-Dutch) and chronic health conditions was obtained at enrollment (Table 2). Child ethnicity (Dutch, non-Dutch) was determined based on the parents’ country of birth: If both parents were born in the Netherlands, the child was classified “Dutch”, and otherwise, the child was classified “non-Dutch” [40]. The prevalence of common chronic conditions was assessed and included asthma, hearing difficulties, seeing difficulties, abdominal pain, headaches or migraine, allergies, and eczema.

**Table 2:** General characteristics of the study population, stratified by children’s weight status (*n* = 3,227)

	Total (*n* = 3,227)	Underweight^a^ (*n* = 449)	Normal weight^a^ (*n* = 2,278)	Overweight^a^ (*n* = 405)	Obesity^a^ (*n* = 50)	Severe obesity^a^ (*n* = 45)
Child characteristics						
Age in years, mean ± SD	5.7 ± 0.4	5.8 ± 0.4	5.7 ± 0.4	5.8 ± 0.4	5.8 ± 0.4	5.9 ± 0.4
Gender**						
% boys	49.3	50.8	51.4	37.0	26.0	66.7
Ethnicity (34 missings)						
% Dutch	98.3	97.3	98.4	99.0	95.9	97.7
BMI**, mean ± SD	15.7 ± 1.7	13.4 ± 0.5	15.45 ± 0.9	18.2 ± 0.6	20.2 ± 0.4	21.7 ± 1.1
Number of chronic conditions, mean ± SD	0.6 ± 0.9	0.6 ± 0.8	0.5 ± 0.9	0.6 ± 0.9	0.7 ± 1.2	0.6 ± 0.8
Number of chronic conditions						
% no chronic condition	62.4	60.9	62.9	61.5	68.0	57.8
Characteristics of the mother						
Age in years, mean ± SD (*n* = 38 missings)	36.5 ± 4.3	36.7 ± 4.0	36.5 ± 4.3	35.3 ± 3.9	36.0 ± 5.2	36.1 ± 6.0
Gender (*n* = 32 missings)						
% mother is respondent	89.3	88.5	89.5	89.3	83.7	93.2
Born in the Netherlands*** (*n* = 32 missings)						
% yes	90.9	91.0	92.0	87.4	79.6	77.3
Educational level*** (*n* = 49 missings)						
% low	3.1	2.3	2.6	4.8	8.2	14.3
% mid-low	17.7	14.9	16.8	23.1	26.5	35.7
% mid-high	46.1	46.8	46.1	47.0	44.9	31.0
% high	33.1	36.0	34.4	25.1	20.4	19.0
Marital status*** (*n* = 46 missings)						
% married or living together	93.5	92.7	94.5	92.1	85.3	80.5
Weight status*** (*n* = 93 missings)						
% normal weight	70.4	81.5	72.5	52.7	47.5	41.0

* *p* < 0.05; ** *p* < 0.01; *** *p* < 0.001: *p* value from chi-square tests for categorical variables and ANOVA for continues variables comparing general characteristics across weight categories

^a^Categories based on international age- and gender-specific BMI cutoff values, for severe obesity cutoff values of BMI: 20.00 in boys and BMI: 21.00 in girls were used ± . According to the World Health Organization definition, BMI < 25: normal weight, BMI 25–30: overweight, BMI > 30: obesity [41]

The majority of the questionnaires were completed by mothers (89.3 %). Information on maternal age (years), height (meters), weight (kilograms), country of birth (Dutch, non-Dutch), educational level (low, mid-low, mid-high, and high) and marital status (married/ cohabiting or single) was obtained at enrollment. Maternal BMI was calculated as weight in kilograms divided by height in meters squared. Weight status of the mother was categorized into one of the two categories based on BMI: no overweight (BMI < 25) or overweight (BMI ≥ 25) [41]. Maternal level of education was categorized into one of the four levels: low (no education, primary school, or ≤3 years of general secondary school), mid-low (>3 years of general secondary school), mid-high (higher vocational training, undergraduate programs, or bachelor’s degree), and high (higher academic education) [42].

### Analyses

Normal weight, overweight, obese, severely obese and underweight children were compared by means of one-way analysis of variance on mean age, average number of chronic health conditions and maternal age. Chi-square tests were performed to compare child (gender, ethnicity, number of chronic health conditions) and maternal characteristics (country of birth, educational level, marital status, weight status) among the weight categories.

Taking into account the non-normal distribution of some of the CHQ-PF28 scale residuals, nonparametric tests were performed to compare scale outcomes across weight categories. The normal weight children were considered the reference group in all analyses. Kruskal–Wallis tests were used to compare the CHQ-PF28 scale scores across weight categories; a significant test (*p* < 0.05) provides statistical support for performing pairwise comparisons [39]. Mann–Whitney U-tests were used to compare the CHQ-PF28 scale scores pairwise; each subgroup was compared with the reference group of normal weight children.

General linear models were fitted to study the association between child weight status and health-related quality of life as measured by the CHQ-PF28 scales. The CHQ-PF28 scales were studied as dependent variables, and the weight categories were studied as determinants, with the normal weight children as reference group. Adjusted general linear models were fitted to correct for potentially confounding variables; both child (age, gender, ethnicity, and number of chronic health conditions) and maternal variables (age, BMI, country of birth, education and marital status) were included. Following the suggestions made by Griffiths et al. [18], an interaction term was introduced to the model to examine whether there were differences in the association between weight status and health-related quality of life for boys and girls and Dutch and non-Dutch children. Associations and interaction terms were evaluated at *p* < 0.05 level. Significant interactions were found for both gender and ethnicity. Statistical analyses were performed with SPSS 18.0 (SPSS Inc., Chicago, IL).

Due to the non-normal distribution of some of the CHQ-PF28 scale residuals, the bootstrap procedure was applied to estimate the regression coefficients of the general linear models [43, 44]. Both the unadjusted (uncorrected for potentially confounding variables) and the adjusted (corrected for potentially confounding variables) bootstrap sampling results are reported with 95 % confidence intervals (95 % CI) and coefficient *p* values. The bootstrap procedure was conducted in R version 2.7.1 (R Development Core Team 2008).

## Results

The mean age of the children in the sample was 5.7 [SD: 0.4] years. Within the sample, 49.3 % were boys. Of all children in the sample, 37.6 % had one or more chronic health conditions. There were significantly more Dutch children in the underweight and normal weight categories compared to the percentage Dutch children in the overweight, obese and severely obese categories (*p* < 0.001). The overall mean BMI of the children in the study sample was 15.7 [SD: 1.7], and the mean BMI of the children with normal weight was 15.5 [SD: 0.9]. There were more boys than girls within the subgroups of severe obesity (66.7 %) and less boys than girls in the overweight and obese subgroups (37.0 and 26 %, respectively). The mean age of the mother was 36.5 years [SD: 4.3], 89.3 % of mothers were born in the Netherlands, and 93.5 % were married or in a de facto relationship (Table 2).

In Table 3, the mean and median scores on the CHQ-PF28 scales are presented. Table 4 presents the bootstrap sampling results of the general linear models.

**Table 3:** CHQ-PF28 scale scores stratified by children’s weight status (*n* = 3,227)

	Normal weight^a,b^ (*n* = 2,278)		Overweight^a^ (*n* = 405)		Obesity^a^ (*n* = 50)		Severe obesity^a^ (*n* = 45)		Underweight^a^ (*n* = 449)		Kruskal–Wallis *p* value^c^
	Mean [SD]	Median [IQR]	Mean [SD]	Median [IQR]	Mean [SD]	Median [IQR]	Mean [SD]	Median [IQR]	Mean [SD]	Median [IQR]	
Physical summary	57.1 [6.1]	58.6 [4.6]	56.3 [7.2]	58.1 [5.5]	56.0 [8.7]	58.1 [6.5]	54.4 [7.3]	56.2 [7.7]**	55.9 [7.3]	58.0 [5.2]***	0.000
Psychosocial summary	53.0 [6.6]	53.8 [8.2]	52.9 [7.3]	54.1 [8.0]	51.4 [7.1]	52.3 [7.3]	52.5 [6.1]	52.9 [6.5]	53.2 [6.3]	53.7 [7.7]	0.552
Physical functioning	97.7 [9.8]	100.0 [0.0]	96.1 [13.6]	100.0 [0.0]*	93.9 [17.0]	100.0 [0.0]***	91.1 [15.8]	100.0 [11.1]***	96.6 [12.2]	100.0 [0.0]	0.000
Role funct.-emo/behav.	97.5 [10.8]	100.0 [0.0]	97.5 [11.5]	100.0 [0.0]	96.0 [14.5]	100.0 [0.0]	96.2 [12.9]	100.0 [0.0]	98.1 [10.0]	100.0 [0.0]	0.432
Role funct.- physical	97.3 [11.1]	100.0 [0.0]	96.6 [13.6]	100.0 [0.0]	95.3 [16.5]	100.0 [0.0]	96.2 [12.9]	100.0 [0.0]	96.1 [13.7]	100.0 [0.0]	0.483
Bodily pain	85.9 [15.7]	80.0 [20.0]	86.7 [15.9]	80.0 [20.0]	82.8 [23.2]	80.0 [20.0]	90.2 [13.4]	100.0 [20.0]	84.1 [17.2]	80.0 [20.0]	0.036
General behavior	71.8 [14.2]	71.3 [18.8]	71.2 [14.9]	71.3 [16.3]	68.3 [13.9]	65.0 [18.8]	72.4 [15.0]	75.0 [18.8]	72.3 [14.1]	71.3 [18.8]	0.287
Mental health	81.8 [14.1]	83.3 [16.7]	81.6 [14.8]	83.3 [16.7]	77.6 [17.3]	83.3 [19.2]	83.7 [13.1]	83.3 [16.7]	81.4 [13.5]	83.3 [16.7]	0.390
Self-esteem	82.1 [13.1]	75.0 [16.7]	82.4 [13.4]	75.0 [25.0]	78.2 [11.9]	75.0 [8.3]*	79.8 [12.4]	75.0 [12.5]	82.4 [13.1]	75.0 [25.0]	0.128
Gen. health perc.	86.1 [14.9]	90.0 [18.8]	83.4 [16.9]	90.0 [22.2]**	78.9 [19.4]	87.5 [25.0]**	78.6 [18.1]	83.8 [31.3]**	83.4 [17.0]	90.0 [25.0]***	0.000
Parental-emotional	89.4 [14.1]	100.0 12.5]	89.7 [15.2]	100.0 [12.5]	84.7 [17.7]	87.5 [25.0]	82.2 [20.4]	87.5 [25.0]**	88.8 [14.5]	87.5 [12.5]	0.010
Parental- time	95.6 [11.9]	100.0 [0.0]	94.0 [16.1]	100.0 [0.0]	94.6 [12.5]	100.0 [0.0]	90.5 [17.8]	100.0 [16.7]*	94.6 [13.4]	100.0 [0.0]	0.070
Family activities	89.3 [16.4]	100.0 [25.0]	89.1 [16.5]	100.0 [25.0]	90.1 [15.9]	100.0 [25.0]	88.9 [15.6]	100.0 [25.0]	88.7 [16.2]	100.0 [25.0]	0.892
Family cohesion	73.5 [17.9]	85.0 [25.0]	73.0 [18.1]	85.0 [25.0]	70.0 [16.9]	60.0 [25.0]	71.1 [17.2]	60.0 [25.0]	74.9 [17.3]	85.0 [25.0]	0.166
Change in health	56.0 [14.9]	50.0 [0.0]	57.1 [17.1]	50.0 [0.0]	57.1 [17.7]	50.0 [0.0]	60.0 [20.2]	50.0 [25.0]	56.3 [15.2]	50.0 [0.00]	0.605

SD standard deviation, *IQR* Interquartile range

* Represents *p* value from Mann–Whitney U test: CHQ-PF28 scale score of each subgroup compared to the scale score of the reference group of normal weight children, * *p* < 0.05; ** *p* < 0.01; *** *p* < 0.001

^a^Subgroups of weight categories based on international age- and gender-specific BMI cutoff values, for severe obesity cutoff values of BMI: 20.00 in boys and BMI: 21.00 in girls were used

^b^Reference group

^c^Kruskal–Wallis tests to compare the CHQ-PF28 scale scores across all subgroups; a significant *p* value (*p* < 0.05) provides statistical support for performing pairwise comparisons

**Table 4:** Associations between children’s weight status and CHQ-PF28 scale scores (*n* = 3,227)

	Overweight^a^ (*n* = 405)	*p* value	Obesity^a^ (*n* = 50)	*p* value	Severe obesity^a^ (*n* = 45)	*p* value	Underweight^a^ (*n* = 449)	*p* value
Physical summary								
Bootstrap unadjusted**	**−0.80 (−1.61; −0.06)**	**0.038**	−1.04 (−3.79; 1.27)	0.424	**−2.72 (−5.14; −0.65)**	**0.008**	**−1.15 (-−.95; −0.42)**	**0.000**
Bootstrap adjusted**	−0.59 (−1.37; 0.17)	0.138	−1.12 (−3.56; 1.27)	0.380	**−2.60 (−4.80; −0.57)**	**0.006**	**−1.11 (−1.85; −0.39)**	**0.002**
Psychosocial summary								
Bootstrap unadjusted	−0.15 (−0.92; 0.66)	0.692	−1.70 (−3.89; 0.30)	0.114	−0.57 (−2.43; 1.27)	0.564	0.22 (−0.45; 0.89)	0.506
Bootstrap adjusted	−0.13 (−0.96; 0.69)	0.734	−1.39 (−4.00; 0.91)	0.280	−0.28 (−2.32; 1.77)	0.794	0.27 (−0.43; 0.95)	0.454
Physical functioning								
Bootstrap unadjusted**	**−1.66 (−3.12; −0.29)**	**0.014**	−3.95 (−9.40; 0.16)	0.062	**−6.71 (−11.47; −2.25)**	**0.000**	−1.16 (−2.40; 0.06)	0.058
Bootstrap adjusted**	**−1.56 (−3.04; −0.08)**	**0.040**	−3.78 (−9.57; 0.96)	0.124	**−5.93 (−10.32; −1.94)**	**0.002**	−1.00 (−2.23; 0.08)	0.060
Role funct.-emo/behav.								
Bootstrap unadjusted	0.03 (−1.19; 1.13)	0.930	−1.46 (−6.00; 1.98)	0.512	−1.34 (−5.88; 1.98)	0.526	0.59 (−0.47; 1.54)	0.268
Bootstrap adjusted	0.07 (−1.40; 1.38)	0.874	−1.37 (−7.05; 2.95)	0.624	−0.49 (−4.96; 3.02)	0.862	0.64 (−0.38; 1.65)	0.246
Role funct.-physical								
Bootstrap unadjusted	−0.69 (−2.21; 0.71)	0.340	−2.00 (−6.66; 1.82)	0.390	−1.19 (−5.20; 2.15)	0.572	−1.15 (−2.52; 0.17)	0.088
Bootstrap adjusted	−0.95 (−2.82; 0.65)	0.234	−2.32 (−7.81; 2.15)	0.396	−1.38 (−5.75; 2.28)	0.544	−1.18 (−2.65; 0.14)	0.082
Bodily pain								
Bootstrap unadjusted*	0.85 (−0.89; 2.40)	0.320	−3.19 (−10.25; 2.87)	0.344	**4.27 (0.09; 8.23)**	**0.048**	**−1.80 (−3.68; −0.09)**	**0.034**
Bootstrap adjusted*	0.84 (−0.93; 2.53)	0.340	−3.15 (−10.58; 3.16)	0.360	1.85 (−2.52; 5.88)	0.400	**−2.42 (−4.28; −0.65)**	**0.008**
General behavior								
Bootstrap unadjusted	−0.66 (−2.23; 0.76)	0.382	−3.60 (−7.51; 0.40)	0.080	0.48 (−4.01; 4.66)	0.808	0.48 (−0.95; 1.87)	0.520
Bootstrap adjusted	−0.56 (−2.29; 1.18)	0.526	−3.28 (7.71; 1.22)	0.156	1.19 (−3.94; 5.81)	0.632	0.28 (−1.29; 1.88)	0.718
Mental health								
Bootstrap unadjusted	−0.24 (−1.75; 1.29)	0.790	−4.30 (−9.29; 0.43)	0.076	2.00 (−2.09; 5.69)	0.324	−0.48 (−1.94; 0.91)	0.488
Bootstrap adjusted	−0.27 (−1.93; 1.45)	0.782	−2.20 (−7.23; 2.81)	0.408	2.39 (−2.04; 6.30)	0.282	−0.03 (−1.52; 1.44)	1.000
Self-esteem								
Bootstrap unadjusted	0.29 (−1.09; 1.59)	0.684	**−3.93 (−7.37; −0.35)**	**0.026**	−2.26 (−5.91; 1.25)	0.228	0.28 (−1.09; 1.58)	0.690
Bootstrap adjusted	0.25 (−1.23; 1.69)	0.736	**−4.18 (−7.87; −0.78)**	**0.012**	−1.25 (−4.96; 2.59)	0.512	0.27 (−1.29; 1.67)	0.712
Gen. health perc.								
Bootstrap unadjusted***	**−2.68 (−4.50; −0.90)**	**0.002**	**−7.06 (−12.55; −1.89)**	**0.006**	**−7.65 (−12.96; −2.58)**	**0.000**	**−2.62 (−4.16; −1.07)**	**0.000**
Bootstrap adjusted**	−1.90 (−3.67; 0.005)	0.052	**−5.39 (−10.33; −0.78)**	**0.018**	−4.41 (−9.91; 0.79)	0.090	**−2.07 (−3.77; −0.45)**	**0.008**
Parental-emotional								
Bootstrap unadjusted*	0.32 (−1.24; 1.89)	0.670	−4.81 (−10.01; 0.20)	0.054	**−7.41 (−13.79; −1.75)**	**0.008**	−0.68 (−2.19; 0.74)	0.378
Bootstrap adjusted	−0.18 (−1.96; 1.60)	0.852	−4.55 (−10.43; 0.71)	0.094	**−6.77 (−13.45; −0.69)**	**0.020**	−0.60 (−2.15; 0.89)	0.450
Parental-time								
Bootstrap unadjusted	**−1.65 (−3.47; −0.08)**	**0.042**	−1.04 (−4.55; 1.92)	0.610	**−5.09 (−10.87; −0.34)**	**0.034**	−1.00 (−2.34; 0.34)	0.160
Bootstrap adjusted	−0.53 (−2.22; 1.04)	0.548	−0.06 (−4.08; 3.32)	0.980	**−5.35 (−11.92; −0.24)**	**0.046**	−1.41 (−2.91; 0.07)	0.060
Family activities								
Bootstrap unadjusted	−0.13 (−1.90; 1.68)	0.870	0.82 (−3.72; 4.93)	0.728	−0.43 (−5.20; 4.02)	0.894	−0.49 (−2.17; 1.17)	0.572
Bootstrap adjusted	−0.004 (−1.86; 1.98)	0.996	1.90 (−3.08; 6.61)	0.434	0.85 (−4.80; 5.55)	0.736	–0.44 (−2.22; 1.26)	0.628
Family cohesion								
Bootstrap unadjusted	−0.55 (−2.60; 1.54)	0.564	−3.45 (−8.01; 0.88)	0.134	−2.62 (−7.30; 2.67)	0.316	1.41 (−0.36; 3.16)	0.116
Bootstrap adjusted	0.07 (−2.08; 2.13)	0.962	−4.43 (−9.31; 0.51)	0.082	−1.56 (−7.34; 4.91)	0.594	0.74 (−1.31; 2.78)	0.504
Change in health								
Bootstrap unadjusted	1.08 (−0.57; 2.91)	0.214	1.20 (−3.38; 6.38)	0.652	3.89 (−1.76; 10.20)	0.202	0.28 (−1.15; 1.80)	0.726
Bootstrap adjusted	0.43 (−1.30; 2.35)	0.642	−0.06 (−5.18; 5.49)	0.970	0.86 (−4.73; 7.21)	0.814	0.15 (1.44; 1.71)	0.832

Bootstrap analyses were performed for the general linear model; values are beta coefficients relative to the normal weight reference group with 95 % confidence intervals. Numbers in boldface indicate a significant beta-coefficient. Unadjusted bootstrap: weight category as independent variable and the CHQ-PF28 scale as dependent variables, no correction for potential confounding variables. Adjusted bootstrap: the model corrected for potential confounding variables; weight category, child characteristics (gender, age, ethnicity, number of chronic conditions) and maternal characteristics (age, country of birth, education level, marital status and overweight yes/no) as independent variables and the CHQ-PF28 scales as dependent variables

* Represents significance level of weight category in the overall bootstrapped model, * *p* < 0.05; ** *p* < 0.01; *** *p* < 0.001

^a^Subgroups of weight categories based on international age- and gender-specific BMI cutoff values, for severe obesity cutoff values of BMI: 20.00 in boys and BMI: 21.00 in girls were used

### CHQ-PF28 summary scales

A significant lower parent-reported score on the physical summary scale for underweight children compared to normal weight children (*p* < 0.01) was found with the general linear model (adjusted model: β, −1.11; 95 % CI, −1.85 to −0.39, *p* < 0.01). Additionally, severely obese children (adjusted model; β −2.60, 95 % CI −4.80 to −0.57, *p* < 0.01) showed lower parent-reported scores on the physical summary scale (Table 3). The general linear model showed no association with lower parent-reported scores on the physical summary scale for overweight and obese children compared to normal weight children (*p* > 0.05). There were no significant differences on the psychosocial summary scale scores between the different weight categories (Table 4).

### CHQ-PF28 profile scales

Parents of overweight, obese and severely obese children reported statistically significant lower scores on the physical functioning scale (*p* < 0.05, *p* < 0.001 and *p* < 0.001 respectively) compared to parents of normal weight children (Table 3); for overweight and severely obese children, this was also found with the general linear model (Table 4).

Parents of obese children reported significant lower scores on self-esteem scales (*p* < 0.05, adjusted model beta, −4.18; 95 % CI, −7.87 to −0.78) (Table 4). Parents of severely obese children reported lower scores regarding the parental impact-emotional and parental impact-time scale (*p* < 0.05) (Table 3).

Obese and underweight children both had lower parent-reported scores on the general health perception scale (*p* < 0.05 and *p* < 0.01 respectively) (Table 3). For underweight children, an association with lower parent-reported scores on the bodily pain (adjusted model: ß −2.42, 95 % CI: −4.28 to −0.65, *p* < 0.01) was found compared to normal weight children (Table 4).

### Effect modification

The physical and psychosocial summary scale showed a significant interaction between weight and child ethnicity (*p* < 0.05); Dutch children showed on average lower parent-reported scores than the non-Dutch children across all weight categories. In similar direction, we found an interaction between weight and child ethnicity (*p* < 0.05) for the physical functioning and the self-esteem scale.

Additionally, a significant interaction was found between weight and gender for the scale of general health (*p* < 0.05); parent-reported scores for boys were on average lower than for girls across weight categories.

## Discussion

Our findings showed lower parent-reported physical health-related quality of life scores of severely obese and underweight children entering elementary school compared to normal weight children. Parent-reported psychosocial health-related quality of life scores were not significantly different across weight categories in the present study.

### Overweight, obesity and health-related quality of life

After adjusting for potential confounding factors such as child gender and child age, the CHQ parent-reported physical summary score was lower among severely obese children. These findings of 5–6-year-old children are in line with findings among adolescents and older school-aged children [9, 11, 14, 15, 19, 20, 27, 45, 46].

The mechanisms underlying the association between BMI and health-related quality of life are not clear yet. It is possible that parents suspect that their overweight children are not able to join their peers in the same level of physical activity, for example during playtime, which thus may contribute to low scores for overweight children on the physical summary scales in our study. It has been hypothesized that physical activity has a BMI-independent positive effect on health-related quality of life [19, 47]. Shoup et al. [10] reported that compared to overweight children who did not meet the recommended physical activity guidelines, older overweight children reported better overall health-related quality of life when they met the recommended physical activity guidelines. Additional analyses (data not shown) in which we studied the association between health-related quality of life and playing outside showed that the amount of outside play (more than 1 h per day) was associated with higher scores on health-related quality of life (*p* < 0.01). However, the association between weight status and health-related quality of life remained statistically significant after adjustment for the amount of outside play (data not shown). So, parents of overweight and obese children report lower health-related quality of life, independent of the level of physical activity of their children.

Our finding that the presence of overweight and obesity in 5–6-year-old children is not associated with the psychosocial summary scale of health-related quality of life-is comparable with the findings reported by others [18, 20, 48]. Decrements to the psychosocial dimension of health-related quality of life seem to become more pronounced during adolescence as young teens become more aware of their physical appearance [25]. Additionally, the use of parent reports might influence the results on psychosocial health-related quality of life in our study. Literature suggests that only few parents realize that their child is overweight or obese at this age [49]; perhaps, parents are therefore not keen on problems that may impact their child’s psychosocial health. On the other hand, parents might ignore psychosocial problems or not take them seriously because they suppose the child is not old enough to be unhappy due to his or her weight.

When exploring the results on the profile scales, it is noteworthy that the parental impact-emotional and parental impact-time scales had significantly lower parent-reported scores for severely obese children. Lower scores can be interpreted as parents having more concerns and less personal time due to their child’s weight, than do parents of normal weight children. This finding has been reported by Wake et al. [20].

### Underweight and health-related quality of life

Parents of underweight children reported lower physical health-related quality of life as compared to parents of normal weight children. These findings are in accordance with previously reported findings among older underweight children and adolescents [20, 24, 27]. Additionally, although analyses were exploratory, significantly more impairment was reported by parents on the bodily pain scale for underweight children. This has been reported only for boys in the study of Wake et al. [20].

We have no explanation for the low health-related quality of life scores reported by parents on the physical domain for underweight young children. In our study, there were no differences in the number of chronic health conditions experienced by underweight children. We have not measured short-term infections such as having the flu or a cold, which might have more impact or influence the amount of energy and physical functioning in underweight children.

There were no impairments reported by the parents of underweight children on the psychosocial dimension, contrary to what has been reported among adolescents [50, 51]. Lower school functioning scores [25] and lower self-esteem scores [11] have been reported among underweight children. We hypothesize that the psychosocial domains of health-related quality of life in young children with overweight may be unaffected or that parents do not recognize impairments on this domain. The impact of lower psychosocial health-related quality of life may have more consequences when children enter early and/or late adolescence.

### Effect modification in the association between health-related quality of life and weight status

On average, parents reported children of “Dutch” origin, relative to “non-Dutch” children, to have lower scores on both the physical and psychosocial summary scales and on the physical functioning and self-esteem scales across all weight categories. The findings for the lower physical scale scores are consistent with previous studies documenting differences among white adolescents and adolescents of African heritage [50]. In the current study, difference in culture between Dutch and non-Dutch parents may have resulted in different reporting. Future research specifying ethnic subgroups instead of a broad ethnic group will have to provide more insight into the relation between ethnicity, weight status and health-related quality of life.

Significantly lower parent-reported scores for boys compared to girls regarding the general health scale were found across all weight categories. Griffiths [18] emphasized gender differences on psychosocial measures of health-related quality of life. However, this review was primarily based on studies among older children. In our study, the use of parent report in combination with the young age of the children might have contributed to a more equal scoring for both genders on the psychosocial and physical health-related quality of life summary scales across all weight categories.

### Strengths and limitations

Some methodological issues are worth noting. Strength of the current study is the large population-based sample available to investigate and compare health-related quality of life of overweight, obese, severely obese and underweight children with normal weight children.

Limitations of the current study include the cross-sectional data that were used to investigate the relationship between weight status and health-related quality of life, and longitudinal research is needed to make any assumptions regarding the causality of the associations found. The choice of confounding variables corrected for in the general linear models was based on the preexisting knowledge about social and biological determinants of weight. Not all potential confounders were measured; for example, no assessment regarding specific conditions or diseases, for example, Coeliac disease [52], was available. The number of common chronic conditions was included as potential confounder. The average number of common chronic conditions was not significantly different between weight categories and was no effect modifier in the association between weight status and health-related quality of life.

The parent form of the CHQ was a feasible measure within this large population-based study. However, the use of parent-proxy reports on a child’s health-related quality of life should be taken into account when interpreting the findings [45, 51, 53, 54].

## Conclusions

This study highlights that as early as elementary school, parents report lower physical health-related quality of life of underweight and severely obese children. Although the decreases in health-related quality of life at this young age are relatively small, they might indicate more decreases in health-related quality of life when children are older. As such, it suggests that nutrition, health education and other interventions must be focused on both parents and children at early ages. Longitudinal studies to evaluate natural and intervention induced developments in body weight and BMI, and the associations with health-related quality of life in childhood are recommended. In the meantime, we recommend physicians, parents and teachers to be aware of the potential negative impact on health-related quality of life in both overweight and underweight children.

## Electronic supplementary material

Below is the link to the electronic supplementary material.

Figure 1 Flow chart of the data collection process in the present study. Supplementary material 1 (TIFF 286 kb)

## Abbreviations

BMI: Body Mass Index
CHQ-PF28: Child Health Questionnaire Parent Form 28
